# Genetic susceptibility in Juvenile Myoclonic Epilepsy: Systematic review of genetic association studies

**DOI:** 10.1371/journal.pone.0179629

**Published:** 2017-06-21

**Authors:** Bruna Priscila dos Santos, Chiara Rachel Maciel Marinho, Thalita Ewellyn Batista Sales Marques, Layanne Kelly Gomes Angelo, Maísa Vieira da Silva Malta, Marcelo Duzzioni, Olagide Wagner de Castro, João Pereira Leite, Fabiano Timbó Barbosa, Daniel Leite Góes Gitaí

**Affiliations:** 1 Department of Cellular and Molecular Biology, Institute of Biological Sciences and Health, Federal University of Alagoas, Maceio, Alagoas, Brazil; 2 Department of Pharmacology, Institute of Biological Sciences and Health, Federal University of Alagoas, Maceio, Alagoas, Brazil; 3 Department of Physiology, Institute of Biological Sciences and Health, Federal University of Alagoas, Maceio, Alagoas, Brazil; 4 Division of Neurology, Department of Neurosciences and Behavioral Sciences, Ribeirão Preto School of Medicine, University of São Paulo, Ribeirão Preto, São Paulo, Brazil; 5 School of Medicine, Federal University of Alagoas, Maceio, Alagoas, Brazil; Odense University Hospital, DENMARK

## Abstract

**Background:**

Several genetic association investigations have been performed over the last three decades to identify variants underlying Juvenile Myoclonic Epilepsy (JME). Here, we evaluate the accumulating findings and provide an updated perspective of these studies.

**Methodology:**

A systematic literature search was conducted using the PubMed, Embase, Scopus, Lilacs, epiGAD, Google Scholar and Sigle up to February 12, 2016. The quality of the included studies was assessed by a score and classified as low and high quality. Beyond outcome measures, information was extracted on the setting for each study, characteristics of population samples and polymorphisms.

**Results:**

Fifty studies met eligibility criteria and were used for data extraction. With a single exception, all studies used a candidate gene approach, providing data on 229 polymorphisms in or near 55 different genes. Of variants investigating in independent data sets, only rs2029461 SNP in GRM4, rs3743123 in CX36 and rs3918149 in BRD2 showed a significant association with JME in at least two different background populations. The lack of consistent associations might be due to variations in experimental design and/or limitations of the approach.

**Conclusions:**

Thus, despite intense research evidence established, specific genetic variants in JME susceptibility remain inconclusive. We discussed several issues that may compromise the quality of the results, including methodological bias, endophenotype and potential involvement of epigenetic factors.

**PROSPERO registration number:**

CRD42016036063

## Introduction

Juvenile Myoclonic Epilepsy (JME) has been recognized by the International League Against Epilepsy (ILAE) as an epileptic syndrome since 1989[1,2] and represents 5% to 10% of all epilepsies[3]. Initial reports indicated JME affects males and females equally, however, recent studies suggest that females outnumber males[4]. The onset of the condition usually occurs in the second decade, ranging from about 8 to 36 years[5]. Although diagnostic criteria differ between epileptologists, it is widely agreed that JME sufferers have early-morning myoclonic seizures (MC) with or without other seizure types (i.e., generalized tonic–clonic seizures and less frequent absences)[2,6,7]. Electroencephalography (EEG) has revealed interictal generalized spike-wave discharges (SWD) and normal background activity for patients with a typical history of JME[8,9]. Patients respond to pharmacological treatment, but with a high recurrence rate on discontinuation of antiepileptic drugs (AEDs)[6,10].

As demonstrated by family and twin studies, genetic factors play a major role in JME[11]. Different heritability models have been used to explain the genetic basis of JME, including Mendelian inheritance of a few major genes or simultaneous involvement of multiple genes with minor effects inherited in non-Mendelian fashion[12,13]. Several methods have been developed over the past 40 years to identify JME causative/susceptibility genes. By using linkage analysis in affected families, researchers have identified genes carrying variations that co-segregate with Mendelian JME (as listed in “Online Mendelian Inheritance in Man”- http://omim.org and http://www.ncbi.nlm.nih.gov/omim/), including *CACNB4* (calcium channel, voltage-dependent, beta 4 subunit)[14], *CASR* (calciumsensing receptor)[15], *GABRA1* (gamma-aminobutyric acid A receptor, alpha 1)[16], *GABRD* (gamma-aminobutyric acid A receptor, delta)[17] and *EFHC1* (EF-hand domain (C-terminal) containing 1[18–20]. Many more chromosome loci have been linked to JME, although their causative genes are still not known[21]. However, it should be noted that these findings only cover a small proportion of JME sufferers[22].

The main hypothesis to explain genetic susceptibility in non-Mendelian JME is based on the interaction among multiple common and/or rare gene variations with modest or strong effects[23,24]. However, the identification of these susceptibility alleles is challenging[25,26]. One widely used experimental approach to investigate common variants is genetic association analysis of candidate genes selected according to their molecular function. Association analyses have mostly been used to assess whether the frequency of specific alleles differs between JME patients and controls more than would be predicted by chance[27]. Although such candidate gene approaches have been useful, they require prior knowledge of gene function.

The completion of the human genome sequences has allowed significant advance in association studies by using unbiased approaches such as genome-wide association studies (GWAS). In the last decade, this strategy has been used to investigate genetic variants associated with several diseases, including epilepsy[28]. Despite the high frequency of information yielded by genetic association studies of JME, the translation of these findings into clinical applications is still limited, requiring a critical appraisal of the existing information. The aim of this systematic review, therefore, was to report and evaluate the findings of existing genetic association studies that have examined the genetic variants underlying the JME phenotype.

## Materials and methods

The systematic review was conducted and reported in accordance with the PRISMA guidelines[29] and the protocol was registered on the international prospective register of systematic reviews (PROSPERO registration number: CRD42016036063. Available at: http://www.crd.york.ac.uk/PROSPERO/display_record.asp?ID=CRD42016036063.

### Search strategy

We did a systematic review to identify genetic association studies with JME. We performed a systematic literature search of PubMed, Embase, Scopus, LILACS, epiGAD (Epilepsy Genetic Association Database), Google Scholar and SIGLE (System for Information on Grey Literature in Europe) up to February 12, 2016 using the following combinations of relevant keywords: “Juvenile Myoclonic Epilepsy” AND “Association Study”, “Juvenile Myoclonic Epilepsy” AND “Polymorphism”, “Idiopathic Generalized Epilepsy” AND “Association Study”, “Generalized Epilepsy” AND “Association Study”, “Juvenile Myoclonic Epilepsy” AND “Variants”, and “Generalized Epilepsy” AND “Variants”.

### Selection criteria

**Fig 1:**
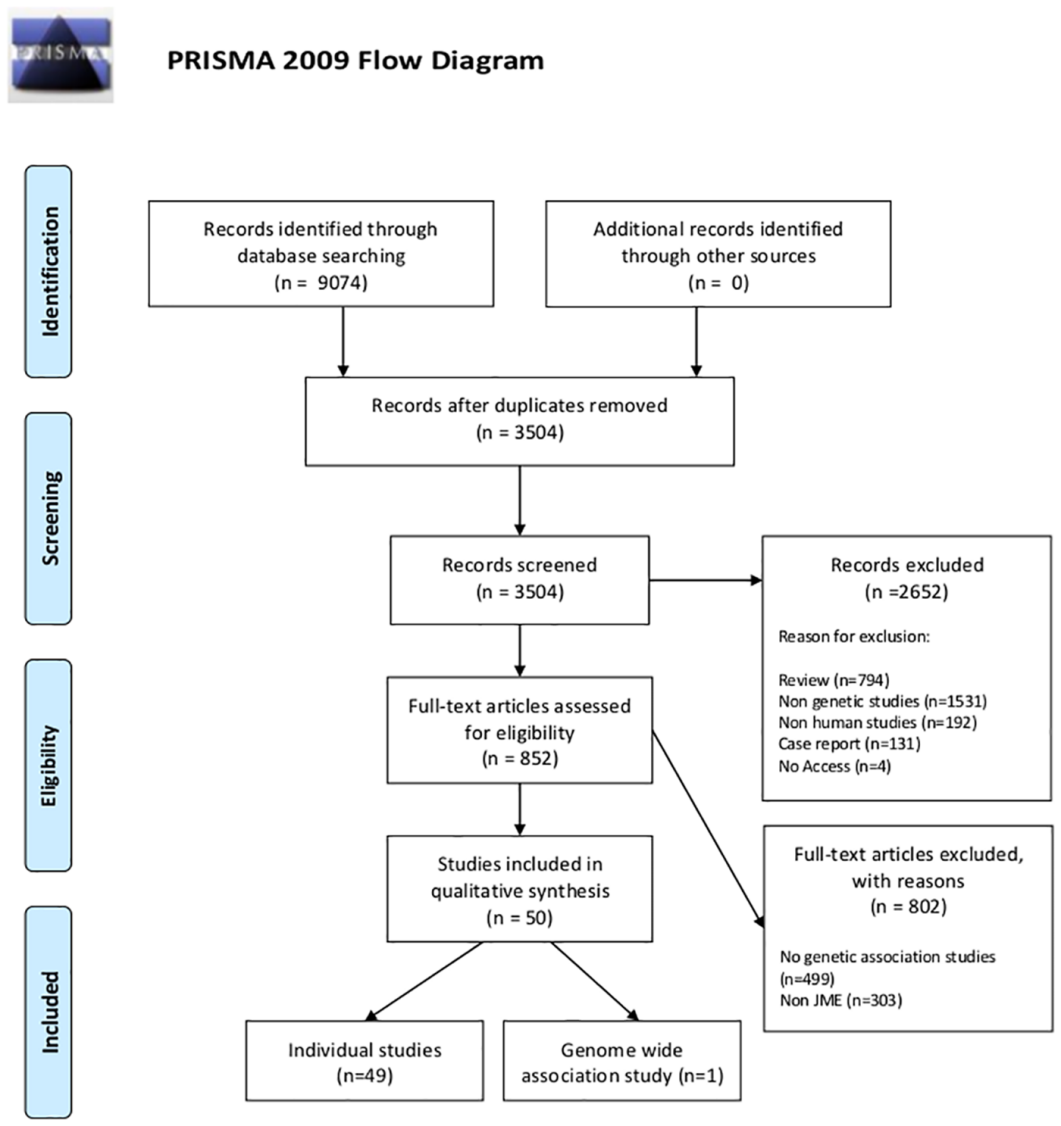
Flow diagram of study identification. *From*: MoherD, Libeiati A, TetzlaffJ, Allman DG, The PRISMA Group {2009). *Preferred Reporting* /terns for Systematic Reviews and *Meta- Analyses*: The PRISMA Statement. PLoS Med 6(7): e1000097. doi:10.1371/journal.pmed1000097. **For more information, visit** www.prisma-statement.org.

We included population-based genetic association studies investigating any polymorphism with JME. Selected articles had to be original research containing independent data and case-control studies, including those that used candidate gene and GWAS approaches. Articles were filtered in three steps (see Fig 1): i) duplicated publications from the databases were excluded; ii) non-relevant studies (based on eligibility criteria) were excluded, such as reviews, non-genetic studies, non-human studies, case reports, and no access; iii): relevant studies were screened to exclude studies conducted with IGE patients without discriminating JME subgroup data and studies with related individuals in case or control groups.

### Data extraction

Two investigators independently (Bruna Santos and Layanne Angelo) performed the literature search and data was cross-checked to ensure consistency. Titles, abstracts, and full texts were screened sequentially for eligibility criteria and any discrepancies were resolved by consensus or by a third reviewer.

Data extracted included information on: i) the setting for each study (the genotyping method employed, the overall sample size and statistical model); ii) characteristics of study participants (phenotypic definitions and ethnic/geographic characteristics); iii) characteristics of polymorphism (type, locus, prior evidence of linkage and evidence of functional role) and; iv) outcome measure (genotype and allele frequencies, Hardy-Weinberg equilibrium test and odds ratio).

### Quality assessment

Methodological quality of the included studies was independently assessed by two reviewers (Bruna Santos and Thalita Marques), according to a set of predefined criteria (S1 Table) based on the scale of Thakkinstian et al.[30], which were amended compared to those used in the previously published meta-analytic studies[31–33]. The following factors were included in the criteria: representativeness of cases, representativeness of control, ascertainment of epileptic disorders, sample size (total number of cases and controls) and matching of case and control participants. Scores ranged from 0 (lowest) to 13 (highest). If the score was ≥7, the study was categorized as “high quality”; otherwise, the study was categorized as “low quality”. Disagreements were resolved by consensus. Due to high heterogeneity in study design and outcome measurements among the included articles, a meta-analysis was not performed. Instead, we conducted a narrative synthesis of the evidence.

## Results

Our search returned 9074 citations, 5570 of which were duplicated. Of the 3504 unique citations, 2652 were excluded because they were not relevant to the current review. Of 852 relevant studies identified, 50 met the predetermined inclusion criteria (Fig 1). Of these, 49 investigated susceptibility variants using a candidate gene approach and one by using GWAS[34–83].

The quality of studies ranged from 5 to 13, out of a possible score of 13 (Table 1 and S2 Table). The most of studies were classified as high quality (90%)[35–53,55,56,58–65,67–74,76–78,80,82,83]. Ninety-eight percent of them clearly define the study population[34–78,80–83]. In relation to representativeness of the controls, fifty-six percent were either population–based or healthy volunteers[36,38–42,45,46,48,51–53,55,58,62–65,67,69–72,74,77,80,82,83] and forty-five percent were both population-based and hospital-based/healthy volunteers/blood donors[34,35,37,43,44,47,49,50,54,56,57,59–61,66,68,73,75,76,78,79,81]. Sixty four percent of control matched only one variable (age, gender or ethnicity) with cases[36–40,42–48,50–54,56,57,62,66,68,69,71,73,75–79,81,82]. Ninety-two percent clearly described diagnosis for JME[35–53,55–78,80,82,83]. Seventy-eight percent of the studies had sample size larger than 200 (number of cases and controls)[38–41,43–68,70–73,76–79,82]. The majority (80%) did not perform genotyping under “blind” conditions (or did not mentioned this aspect). Results of HWE analysis were reported in 70% of the studies[36–41,43,45–47,49–53,55,56,58–60,62–65,67–72,74,76–78,82]. Ninety percent of the studies assessed the association between genotypes and JME using X^2^ test and logistic regression, according to Clarke et al.[84][34–39,41–53,55–68,70–78,80–82].

**Table 1:** Polymorphisms investigated in independent studies.

Gene	Locus	Previous evidence of linkage with JME	SNP	JME/Control	Association	Population	Ethnicity control	Quality scolre	Study
CX36	15q14	JME (OMIM 604827)	rs3743123 (C588T)	247/621	Yes	German	PB+GC	12	Hempelmann, 2006 [52]
				140/123	Yes	European	PB	11	Mas, 2004 [47]
GRM4	6p21	JME (OMIM 608816)	rs2029461 G/A	249/186	Yes	Indian	PB+FB	12	Parilhar, 2014 [67]
				215/732	Yes	German	PB	11	Muhle, 2010 [62]
BRD2	6p21	JME (OMIM 608816)	rs3918149	20/64	Yes	North American	PB+FB	7	Pal, 2003[83]
				34/256	Yes	European	PB	12	Cavalleri, 2007 [55]
				57/227	Yes	Irish	PB	12	Cavalleri, 2007 [55]
				159/154	No	West European	PB	11	Layouni, 2010 [60]
				48/144	No	Southern Indian	PB	12	Cavalleri, 2007 [55]
				146/99	No	Australian	PB	12	Cavalleri, 2007 [55]
				246/664	No	German	PB	12	Cavalleri, 2007 [55]
CHRNA4	20q13.33	Other epilepsy (OMIM 118504)	c.594C>T	92/137	No	Polish	PB	12	Rozycka, 2009 [58]
				60/94	No	German	PB	9	Steinlein, 1997 [37]
				<50/198	No	Caucasian (UK)	PB	9	Chioza, 2002b [44]
			1674(+14)A>G	92/137	No	Polish	PB	12	Rozycka, 2009 [58]
				<50/198	No	Caucasian (UK)	PB	9	Chioza, 2002b [44]
				60/94	No	German	PB	9	Steinlein, 1997 [37]
			T1545C	60/94	No	German	PB	9	Steinlein, 1997 [37]
				<50/198	No	Caucasian (UK)	PB	9	Chioza, 2002b [44]
GABRB3	15q12	Other epilepsy (OMIM 137192)	rs4906902	44/180	No	Australian	PB	5	Heron, 2007 [79]
				304/561	No	German	PB+GC	10	Hempelmann, 2007 [56]
GRM4	6p21	JME (OMIM 608816)	rs937039 G/A	215/732	No	German	PB	11	Muhle, 2010 [62]
				249/186	No	Indian	PB+FB	12	Parilhar, 2014 [67]
			rs745501 T/A	215/732	No	German	PB	11	Muhle, 2010 [62]
				249/186	No	Indian	PB+FB	12	Parilhar, 2014 [67]
			rs2451334 T/C	215/732	No	German	PB	11	Muhle, 2010 [62]
				249/186	No	Indian	PB+FB	12	Parilhar, 2014 [67]
			rs2499697 C/A	249/186	No	Indian	PB+FB	12	Parilhar, 2014 [67]
				215/732	No	German	PB	11	Muhle, 2010 [62]
KCNN3 (hSkCa3, hKCa3)	1q21.3	No	CAG20	78/290	No	German	PB+FB	11	Sander, 1999 [40]
				222/248	No	South India	PB	10	Vijai, 2005 [48]
			CAG21	78/290	No	German	PB+FB	11	Sander, 1999 [40]
				222/248	No	South India	PB	10	Vijai, 2005 [48]
TAP1	6p21	JME (OMIM 608816)	Ile333Val	14/81	No	Tunisian and European	PB	9	Layouni, 2010b[61]
				159/154	No	West European	PB	11	Layouni, 2010 [60]
			Asp637Gly	154/159	No	Tunisian and European	PB	9	Layouni, 2010b [61]
				159/154	No	West European	PB	11	Layouni, 2010 [60]
HLA	6p21	JME (OMIM 608816)	DQB1*0603	93/93	No	European	PB	7	Le Hellard, 1999 [75]
				24/129	No	Scandinavian	PB	6	Moen, 1995 [81]
BRD2	6p21	JME (OMIM 608816)	rs516535	20/64	Yes	North American	PB+FB	7	Pal, 2003[83]
				159/154	No	West European	PB	11	Layouni, 2010 [60]
				102/360	No	Dutch	PB	6	de Kovel, 2007 [54]
GABRG2	5q34	JME (OMIM 137164)	rs211037 (Asn196Asn)	201/267	Yes	Indian	PB	12	Balan, 2013 [64]
				98/130	No	Brazilian (Alagoas)	PB	13	Gitaí, 2012 [63]
HLA	6p21	JME (OMIM 608816)	DQB1* 0603 and 0604	24/24	Yes	European	PB	6	Greenberg, 1996 [34]
				93/93	No	European	PB	7	Le Hellard, 1999 [75]
HLA	6p21	JME (OMIM 608816)	DRB1* 1301 and 1302	62/77	No	German	PB	10	Sander, 1997 [36]
				93/93	No	European	PB	7	Le Hellard, 1999 [75]
				24/24	Yes	European	PB	6	Greenberg, 1996 [34]
KCNJ10	1q23.2	No	rs1130183	124/284	No	Chinese	PB	9	Guo, 2015 [73]
				218/660	Yes	German	PB	12	Lenzen, 2005 [51]

**Abbreviations** SNP, single nucleotide polymorphism; JME, Juvenile Mioclonic Epilepsy; BRD2, Bromodomain Containing 2; CHRNA4, cholinergic receptor, nicotinic alpha 4; CX36, connexin-36; GABRB3, gamma-aminobutyric acid type A receptor beta3 subunit; GRM4, glutamate receptor, metabotropic 4; KCNN3, potassium channel, calcium activated intermediate/small conductance subfamily N alpha, member 3; TAP1, transporter 1, ATP-binding cassette; GABRG2, gamma-aminobutyric acid (GABA) A receptor, gamma 2; HLA-DQB1, major histocompatibility complex, class II, DQ beta 1; HLA-DRB1, major histocompatibility complex, class II, DR beta 1; KCNJ10, potassium channel, inwardly rectifying subfamily J, member 10; PB: Population-based; FB: Family-based; GC: Genomic control.

### Gene candidate studies

In all, 49 published studies provided data regarding 224 polymorphisms in or near 52 different genes, of which 33 were directly related to synapse transmission (channels, receptors, neurotransmitters and neuromodulators). The others were involved in different biological processes, such as gene expression regulation, mitochondrial metabolism and immunological response (S2 Table).

The studies included in the review were conducted with different ethnic populations from Europe (n = 34), America (n = 5), Asia (n = 10), Africa (n = 2) and Oceania (n = 3). The number of patients ranged from 14 to 732, and their age varied from 2 to 25 years. The most used JME diagnostic criterion was based on the proposal by the Commission on Classification and Terminology of the International League Against Epilepsy. The vast major of polymorphisms failed to show associations with JME (S2 Table).

Twenty-two polymorphisms were investigated, independently, in more than one study. For 14 polymorphisms, all independent investigations showed no association (Table 1). Only rs2029461 SNP in *GRM4*, rs3743123 in *CX36* and rs3918149 in *BRD2* showed a significant association with JME in at least two different background populations. For 5 polymorphisms, the positive association was not confirmed in independent studies (Table 1). For example, rs516535 in *BRD*2, which had reported analysis in several background populations, showed a significant association with JME in Northern American population[83], but no association in larger samples of West European[54,60].

### GWAS studies

Only one study involved a genome-wide analysis of JME patients. The EPICURE study published a large GWAS in GGE, including 382 JME patients of North-Western European origin and 382 ethnically matched population controls. By combined analysis of the 2-stage, only SNP rs12059546 in M3 muscarinic acetylcholine receptor (*CHRM3*), reached genome-wide significance with JME (S2 Table). Furthermore, only 10 SNPs located at 8 different loci (1q43; 3q21.31; 5q12.3; 8q23.1, 11p15.4, 13q13.2, 18q11.2, 18q22.3) showed associations with JME exceeding the Stage-1 screening threshold of PLMM < 1.0 × 10−5 and none of them are among those included in this review.

## Discussion

To the best of our knowledge, this is the first systematic review of genetic association studies in JME. Our review provides an updated perspective on the accumulating evidence on common susceptibility alleles in this IGE subtype. In the 50 association studies reviewed, most polymorphisms were examined in one case–control study, of which just 17% had a positive association[34,43,47–49,51–53,55,58,61,62,64,66–69,71,77,80–83]. However, taking into account the high *a priori* risk of false positive results in candidate gene association studies[25], a discussion of the biological significance of these cases was precluded. In fact, genetic associations based on a single study cannot exclude the possibility of having been obtained by chance, and thus are not sufficient to establish a link with JME susceptibility. The rest of the discussion is therefore limited to data generated in more than one independent study.

Positive findings using variants from independent data sets could not be replicated in at least one of the studies, including *GABRG2* (rs211037), *HLA* (DRB1), *HLA* (DQB1), *BRD2* (rs3918149 and rs516535), *KCNJ10* (rs1130183). As we discussed below, part of the reason for the lack of consistent patterns of association could be the experimental design: sample size, population stratification and phenotype definition.

Sample size: the recruitment of sufficiently large and homogeneous samples for robust genetic analysis is a long-standing weakness of association studies[25,85,86]. The using of small sample size reduces the statistical power to detect loci with a positive effect. On the other hand, larger sized samples may be more heterogeneous as a result of an effort to get larger cohorts. Population stratification: studies with discrepant results were often conducted on patients with different population backgrounds. For example, *GABRG2* (rs211037) had a significant association with an Indian population but not in a Brazilian population. Interestingly, the allele and genotype frequencies of these polymorphisms show wide variation between the populations investigated, suggesting a role for ethnic differences in the distribution of this variant[63]. In these cases, the lack of replication could be caused by differences in the genetic structure of populations investigated. Population stratification could exist between treatment and control populations, even in well-designed studies. Such stratification could lead to spurious associations between a disease and genes that are biologically unrelated to the disease. In almost all the studies included in our sample, the only method to minimize stratification was by sampling and matching cases and controls from the same geographic region. Only four studies applied a complementary method by using genetic markers[52,53,56,57]. Thus, undetected population stratification could also be a cause of non-replicable studies[87–89], especially if the variant studied has variable penetrance and allele frequencies in different populations[90].

Phenotype definition: the lack of diagnosis based on rigid standards or objective biomarkers is a critical issue in the genetic analysis of JME[12] and may explain the divergent results found in this study. Most of the studies classified patients according to those suggested by the Commission on Classification and Terminology of the International League Against Epilepsy[2] in their Proposal for Revised Classification of Epilepsies and Epileptic Syndromes from 1989. In this document, the League described a group of signs and symptoms to identify a JME patient, but did not establish a “diagnostic protocol. Thus, even though most researchers followed the ILAE clinical criteria, inconsistent interpretation of clinical parameters and electrographic findings could still contribute to the divergent results. For example, Balan et al.[71] only used abnormal findings on EEG recordings to support JME diagnosis, while Gitaí et al.[63] included generalized spike-wave discharges in their diagnosis. Moreover, JME is a heterogeneous electroclinical epilepsy syndrome[91,92]. Few studies have used a tight endophenotype criterion, grouping patients by seizure type, diurnal preferential seizure occurrence or electroencephalogram pattern. Thus, the clinical entities classified as JME display many differentiable symptoms[93] that may well reflect different underlying genetic influences. There is a subset of JME patients, for example, who evolved from childhood absence epilepsy (CAE)[6]. If samples are not divided into subclinical categories, the genetic signal may be masked. A more effective strategy to elucidate genetic markers associated with JME could be to narrowly and consistently identify phenotypes representing specific JME endophenotypes [94–97].

Thus, because of the difficulty in controlling genetic heterogeneity and all possible confounders across studies, the failure of replication does not prove a false-positive result. Although independent replication of association has been a normative criterion for weighing evidence, Pal et al.[98] suggest that evidence should also be judge by integrating results from different experimental approaches, including linkage analysis and mutation screening. Indeed, a positive allelic association found in a locus of prior linkage is more likely to be real[98,99]. Returning to the case of variants in *BRD2*, EJM1, a major JME susceptibility locus, was discovered by linkage analysis of three separate family collection[36,100–103]. In 2003, Pal et al.[83] suggested that *BRD2* is responsible for the EJM1 linkage peak and that the rs3918149 (among others) variant is a risk factor for JME. The positive association of this variant with JME was confirmed by independent familial and populational-based case-control studies[77,83]. Furthermore, *BRD2* (but not rs3918149) was associated with photoparoxysmal response (PPR)[104]. Therefore, although the relationship between *BRD2* and JME has not been replicated across some populations[54,60,77] convergent evidence supports *BRD2* contributions to epileptogenesis. In fact, functional assays with heterozygous *BRD2* knockout mice showed an increase in seizure susceptibility to flurothyl and the occurrence of spontaneous seizures in female mice[105]. In this review, out of 39 variants with positive associations, 23 are located in areas linked to JME. An absence of replication for these polymorphisms, therefore, should not prevent their incorporation in functional studies.

Beyond the rs3918149 in *BRD2*, only two other polymorphisms showed significant associations with JME (rs2029461 in *GRM4* and rs3743123 in *CX36*) which were replicated in at least one independent study. In fact, these studies showed higher quality scores. For example, to avoid the confounding effect of population admixture in case-control studies, at least, one of these studies applied a genomic control approach[52] or carried out a family-based association study in parent–child-trios[46,67,83]. *CX36* is an integral membrane protein of neuronal gap junction channels that has a significant role in epileptogenesis[106–108]. rs3743123 is a C.T transition (c.588C.T) within exon two that has not been classified as biologically important. Two independent studies showed that subjects with the T/T genotype at position 588 had a significantly increased risk of JME in a German population (OR 4.3; 95% CI 1.49 to 12.3) and a mix of other European (OR1.62; 95% CI 1.02–2.57) populations. The *GRM4* encoding the group III metabotropic glutamate receptor 4 (mGluR4) and several studies have indicated a functional importance for this gene in the genesis of epilepsy[109–111]. rs2029461 is an A/G change located in the 5`UTR. The minor allele (G) showed significant association with the JME phenotype in both Caucasian and Indian populations. Interestingly, both *CX36* and *GRM4* genes are located in two major susceptibility loci (EJM2) for JME: regions 15q14 and 6p21, respectively, and were therefore originally chosen as gene candidate due to positional and functional criteria. However, the mechanisms by which rs3743123 and rs2029461 predispose individuals to developing JME remain obscure.

### JME susceptibility

Despite intense research over the last decades, there is relatively weak evidence for the involvement of most of the variants investigated in JME susceptibility. Even in a more systematic investigation by using GWAS, the findings are not particularly encouraging. In fact, the single GWAS study only identified rs12059546 (located in the gene encoding the M3 muscarinic acetylcholine receptor (*CHRM3*)) as having genome-wide significance with JME. However, this positive association was not replicated in a case/control study performed in a Chinese population[112].

This apparent lack of progress may be caused by several confounding issues, including the paradigm that epilepsy is a channelopathy[113]. We observed that the majority of candidate gene studies (64%) had investigated variants in gene coding ion channels or proteins directly related to synapses transmission. These findings clearly indicate that the search for JME related genes has been narrowed by the assumption that the underlying cause of epilepsy is channel gene dysfunction. However, it is highly likely that epilepsies result from an interaction between genetic variants with different functional roles. A recent study using exome sequencing followed by large-scale genotyping of individuals with IGE provided a candidate list of epilepsy-susceptibility variants that was not limited to genes encoding ion channels or ion channel modifiers[114]. Clearly, further studies are necessary to confirm that these variants are genuinely contributing to JME susceptibility.

Although individual or genome-wide association analyses offer a powerful strategy for identifying common variants of a complex disease, such as JME, major influences on disease expression caused by rare alleles are often missed. Advances in genomic technologies can expand our understanding of the genetics of JME[95]. For example, Mefford et al.[94] detected several rare copy number variants (CNVs) in JME patients as well as in several other epilepsy types by using whole-genome oligonucleotide array comparative genomic hybridization still a lack evidence of causality between these variants and JME.

Other studies have showed that many of these genomic structural variants are potential risk factors for JME, but are present only in 3% of patients[96]. With the advent of next-generation sequencing technologies (NGS) that allow whole-genome or whole-exome sequencing, there will be an unprecedented increase in the identification of multiple rare DNA variations that may be associated with particular phenotypes[97]. However, to date, the only NGS study of individuals with JME suggests that moderately rare variants (frequency range of 0.06%–0.3%) with intermediate effects do not play a significant role in JME risk or the development of other IGE subtypes. Moreover, no single rare variant was detected exclusively in JME patients that could account for more than 1% of cases. This high genetic heterogeneity might help explain the numerous unsuccessful attempts to find JME susceptibility genes. Alternatively, JME heritability could be epigenetic, including changes in methylation patterns of genome and histones. Such changes could affect susceptibility to and development/maintenance of epilepsy. In fact, the detection of epigenetic modifications observed in both animal models and tissues from patients with temporal lobe epilepsy are encouraging a new line of research that may contribute substantially to our knowledge of epilepsy susceptibility[115]. The significant challenge is how to apply these approaches to investigate risk factors in IGE epilepsy, such as JME.

## Conclusions

Considerable effort has been expended over the last 40 years to identify JME causative/susceptibility genes. Here, we provided an updated synthesis of the accumulating findings of genetic association studies and JME. The combined studies provided data on 229 polymorphisms in (or near) 55 different genes. Nevertheless, only three polymorphisms (rs2029461 SNP in *GRM4*; rs3743123 in *CX36* and rs3918149 in *BRD2*) have been associated with JME in, at least, two independent gene candidate investigations. The lack of success in replicating the results is related to various aspects, including limitations of experimental design, endophenotypes, channelopathy issues and genetic heterogeneity. Therefore, scientists should go beyond replication criteria and draw on convergent evidence across different study designs. Such an integration of results from different experimental approaches combined with epigenetics and genomic technology could lead us to a more comprehensive evaluation of the current state of JME susceptibility.

## Supporting information

S1 Table Scale for quality assessment of genetic association studies of epileptic disorders. (DOCX)

S2 Table Characteristics of the studies included in the systematic review. (DOC)

S1 Checklist PRISMA 2009 checklist. (DOC)

S1 File Meta-analysis on genetic association studies checklist. (DOCX)

S2 File List the excluded articles. (XLSX)
