# Supplementary material for: Genetic susceptibility in Juvenile Myoclonic Epilepsy: Systematic review of genetic association studies
Source: PLoS One. 2017 Jun 21;12(6):e0179629. doi: 10.1371/journal.pone.0179629 (PMC5479548; doi:10.1371/journal.pone.0179629)
Supplement: S1 Table — (DOCX) [file pone.0179629.s001.docx]

**S1 Table. Scale for Quality Assessment of genetic association studies of epileptic disorders**

| **Quality parameters** | **Score** |
| --- | --- |
| **A - Representativeness of case** |  |
| Consecutive/randomly selected from case population with clearly defined sampling frame | 2 |
| Consecutive/randomly selected from case population without clearly defined sampling frame | 1 |
| No method of selection described | 0 |
| **B - Representativeness of control** |  |
| Population-based or healthy volunteers | 2 |
| Both population-based and hospital-based/healthy volunteers/blood donors | 1 |
| Not described | 0 |
| **C - Ascertainment of epileptic disorders** |  |
| Clearly described objective criteria for diagnosis of Juvenile Myoclonic Epilepsy | 1 |
| Not described | 0 |
| **D - Sample size (total number of cases and controls)** |  |
| Larger than 200 | 2 |
| Larger than 100, but less than 200 | 1 |
| Less than 100 | 0 |
| **E - Genotyping examination** |  |
| Genotyping done under "blinded" condition | 1 |
| Unblinded or not mentioned | 0 |
| **F - Hardy-Weinberg equilibrium** |  |
| Hardy-Weinberg equilibrium | 2 |
| Hardy-Weinberg disequilibrium | 1 |
| Not mentioned | 0 |
| **G - Association of assessment** |  |
| Assess association between genotypes and epileptic disorders with appropriate statistics and adjustment for confounders | 1 |
| Inappropriate statistics used | 0 |
| **H - Matching of case and control participants** |  |
| Controls matched with cases more than one variable (i.e., age, gender and ethnicity) | 2 |
| Controls matched with cases only one variable (i.e., age, gender or ethnicity) | 1 |
| Not matched or not descried | 0 |
| **Total** | 13 |

Studies were categorized as ‘‘high quality’’ if the quality score was ≥7; otherwise, studies were categorized as ‘‘low quality’’
