# Supplementary material for: Genetic susceptibility in Juvenile Myoclonic Epilepsy: Systematic review of genetic association studies
Source: PLoS One. 2017 Jun 21;12(6):e0179629. doi: 10.1371/journal.pone.0179629 (PMC5479548; doi:10.1371/journal.pone.0179629)
Supplement: S2 Table — (DOC) [file pone.0179629.s002.doc]

**S2 Table. Characteristics of the studies included in the Systematic Review.**

| **Study** | **Population** | **Gene** | **Polymorphisms** | **Methods** | **Cases / Controls** | **Association** | **Quality score** | **Stratification control** | **Locus** | **Previous evidence of linkage with JME** |
| --- | --- | --- | --- | --- | --- | --- | --- | --- | --- | --- |
| Greenberg, 1996 [34] | European | HLA | HLA-DRB1 | PCR | 24/24 | Yes | 6 | PB | 6p21 | JME  OMIM 608816 |
|  |  |  | HLA-DQB1 |  |  | Yes |  |  |  |  |
|  |  |  | HLA-DPB1 |  |  | No |  |  |  |  |
| Guipponi, 1997 [35] | European | GABRA5 | Dinucleotide repeat polymorphisms | PCR | 88/100 | No | 8 | PB | 15q12 | other epilepsy  OMIM 137192 |
|  |  | GABRB3 | Dinucleotide repeat polymorphisms |  |  | No |  |  |  |  |
| Sander, 1997 [36] | German | HLA | DR13 (DRB1) | PCR | 62/77 | No | 10 | PB | 6p21 | JME  OMIM 608816 |
| Steinlein, 1997 [37] | German | CHRNA4 | 145−55/HaeIII | PCR/SSCA | 60/94 | No | 9 | PB | 20q13.33 | other epilepsy  OMIM 118504 |
|  |  |  | 594/CfoI |  |  | No |  |  |  |  |
|  |  |  | 1545/CfoI |  |  | No |  |  |  |  |
|  |  |  | 1674 + 14/StyI |  |  | No |  |  |  |  |
| Sander, 1999 [38] | German | PAX6 | PAX-6LPR | PCR | 70/354 | No | 11 | PB | 11p13 | other epilepsy  OMIM 117100 |
| Sander, 1999 [39] | German | GABABR1 | Ala20Val | PCR-RFLP | 72/130 | No | 11 | PB | 6p22.1 | No associated |
|  |  |  | Gly489Ser |  |  | No |  |  |  |  |
|  |  |  | T1974C |  |  | No |  |  |  |  |
| Sander, 1999 [40] | German | hKCa3 (KCNN3) | CAG20 | PCR | 78/290 | No | 10 | PB+FB | 1q21.3 | other epilepsy  OMIM 118507 |
|  |  |  | CAG21 |  |  | No |  |  |  |  |
|  |  |  | CAG22 |  |  | No |  |  |  |  |
|  |  |  | CAG23 |  |  | No |  |  |  |  |
|  |  |  | CAG24 |  |  | No |  |  |  |  |
|  |  |  | CAG25 |  |  | No |  |  |  |  |
|  |  |  | CAG26 |  |  | No |  |  |  |  |
|  |  |  | CAG27 |  |  | No |  |  |  |  |
|  |  |  | CAG28 |  |  | No |  |  |  |  |
|  |  |  | CAG29 |  |  | No |  |  |  |  |
|  |  |  | CAG30 |  |  | No |  |  |  |  |
|  |  |  | CAG31 |  |  | No |  |  |  |  |
|  |  |  | CAG32 |  |  | No |  |  |  |  |
|  |  |  | CAG33 |  |  | No |  |  |  |  |
|  |  |  | CAG34 |  |  | No |  |  |  |  |
|  |  |  | CAG35 |  |  | No |  |  |  |  |
|  |  |  | CAG36 |  |  | No |  |  |  |  |
|  |  |  | CAG37 |  |  | No |  |  |  |  |
| Haug, 2000 [41] | German | MAO-A | MAO-A | PCR | 126/248 | No | 12 | PB | Xp | No associated |
| Sobetzko, 2001[42] | German | GLRA3 | T240C | PCR | 61/116 | No | 9 | PB | 4q34.1 | No associated |
|  |  |  | C243T |  | 61/116 | No |  |  |  |  |
|  |  |  | C876T |  | 41/141 | No |  |  |  |  |
|  |  |  | G1472A |  | 40/134 | No |  |  |  |  |
| Chioza, 2002 [43] | Caucasians | CACNA1A | Int6a | PCR, DHPLC and automated sequencing | 232/234 | No | 10 | PB+FB | 19p13.13 | other epilepsy  OMIM601011 |
|  |  |  | Int6b |  |  | No |  |  |  |  |
|  |  |  | Int6c |  |  | No |  |  |  |  |
|  |  |  | Int7a |  |  | No |  |  |  |  |
|  |  |  | Int7b |  |  | Yes |  |  |  |  |
|  |  |  | EX8 |  |  | No |  |  |  |  |
|  |  |  | Int8a |  |  | No |  |  |  |  |
|  |  |  | Int8b |  |  | No |  |  |  |  |
|  |  |  | Int10a |  |  | No |  |  |  |  |
|  |  |  | Int10b |  |  | No |  |  |  |  |
|  |  |  | Int11 |  |  | No |  |  |  |  |
| Chioza, 2002 [44] | Caucasians (UK) | KCNJ3 | T1038C | PCR-RFLP | <50/198 | No | 9 | PB | 2q24.1 | No associated |
|  |  |  | T1505(+1596Ab) |  |  | No |  |  |  |  |
|  |  | KCNJ6 | G495A |  |  | No |  |  | 21q22.13 | No associated |
|  |  |  | T1032C |  |  | No |  |  |  |  |
|  |  | KCNQ2 | C1419G |  |  | No |  |  | 20q13.33 | other epilepsy  OMIM 602235 |
|  |  |  | T2154A |  |  | No |  |  |  |  |
|  |  |  | C2255A |  |  | No |  |  |  |  |
|  |  | CHRNA4 | C594T |  |  | No |  |  | 20q13.33 | other epilepsy  OMIM 118504 |
|  |  |  | T1545C |  |  | No |  |  |  |  |
|  |  |  | A1674(+14G) |  |  | No |  |  |  |  |
| Sander, 2002 [45] | German | CACNA1A | 1467 G→A, Glu394 (SNP8) | Pyrosequencing | 139/186 | No | 11 | PB+FB | 19p13.13 | other epilepsy  OMIM 601011 |
| Izzi, 2003 [46] | German | GRM4 | rs2499679 | Pyrosequencing | 144/144 | No | 11 |  | 6p21 | JME  OMIM 608816 |
| Mas, 2004 [47] | European | CX36 | rs651724 | Pyrosequencing | 169/123 | No | 11 | PB | 15q14 | JME  OMIM 604827 |
|  |  |  | 369C>T |  |  | No |  |  |  |  |
|  |  |  | rs3743123 |  |  | Yes |  |  |  |  |
|  |  |  | 888G>A |  |  | No |  |  |  |  |
|  |  |  | rs2277558 |  |  | No |  |  |  |  |
| Vijai, 2005 [48] | South India | hSKCa3 | CAG13 | PCR | 222/248 | No | 10 | PB | 1q21.3 | No associated |
|  |  |  | CAG14 |  |  | No |  |  |  |  |
|  |  |  | CAG15 |  |  | No |  |  |  |  |
|  |  |  | CAG16 |  |  | Yes |  |  |  |  |
|  |  |  | CAG17 |  |  | No |  |  |  |  |
|  |  |  | CAG18 |  |  | Yes |  |  |  |  |
|  |  |  | CAG19 |  |  | Yes |  |  |  |  |
|  |  |  | CAG20 |  |  | No |  |  |  |  |
|  |  |  | CAG21 |  |  | No |  |  |  |  |
|  |  |  | Agg |  |  | No |  |  |  |  |
| Gu, 2005 [49] | German | EFHC2 | rs5906926 C/G | Taqman | 245/661 | No | 12 | PB | XP11.4 | No associated |
|  |  |  | rs1885293 G/T |  | 246/659 | No |  |  |  |  |
|  |  |  | rs9887166 A/G |  | 245/659 | No |  |  |  |  |
|  |  |  | rs2208592 G/T |  | 244/658 | Yes |  |  |  |  |
|  |  |  | rs1181064 C/T |  | 246/658 | No |  |  |  |  |
|  |  |  | rs1448865 C/T |  | 246/658 | No |  |  |  |  |
| Lenzen, 2005 [50] | German | GABRD | Arg220His (659G>A ) | TaqMan | 218/664 | No | 11 | PB | 1p36.33 | JME  OMIM 613060 |
| Lenzen, 2005 [51] | German | KCNJ10 | Arg271Cys (rs1130183) | TaqMan | 218/660 | Yes | 12 | PB | 1q23.2 | No associated |
| Hempelmann, 2006 [52] | German | CX36 | rs3743123 (C588T) | TaqMan | 247/621 | Yes | 12 | PB+GC | 15q14 | JME  OMIM 604827 |
| Lorenz, 2006 [53] | German | ALDH5A1 | rs1883415 | Taqman | 217/651 | No | 11 | PB+GC | 6p22 | other epilepsy  OMIM 608072 |
|  |  |  | rs2760118 |  | 216/655 | No |  |  |  |  |
|  |  |  | SNPex4 |  | 217/662 | No |  |  |  |  |
|  |  |  | rs2252525 |  | 218/661 | Yes |  |  |  |  |
|  |  |  | rs807616 |  | 218/661 | No |  |  |  |  |
|  |  |  | rs2247845 |  | 218/663 | No |  |  |  |  |
|  |  |  | M06TS13 |  | 201/632 | No |  |  |  |  |
| de Kovel, 2007 [54] | Netherlands | BRD2 | rs516535_C | TaqMan | 102/360 | No | 6 | PB | 6p21 | JME  OMIM 608816 |
|  |  |  | rs206781_T |  |  | No |  |  |  |  |
|  |  |  | rs188245_G |  |  | No |  |  |  |  |
| Cavalleri, 2007 [55] | European | BRD2 | rs3918149 | TaqMan | 34/256 | Yes | 12 | PB | 6p21 | JME  OMIM 608816 |
|  | Irish |  |  |  | 57/227 | Yes |  |  |  |  |
|  | South India |  |  |  | 48/144 | No |  |  |  |  |
|  | Australian |  |  |  | 146/99 | No |  |  |  |  |
|  | German |  |  |  | 246/664 | No |  |  |  |  |
| Hempelmann, 2007 [56] | German | GABRB3 | rs4906902 (−897 T > C) | TaqMan | 304/561 | No | 10 | PB+GC | 15q12 | other epilepsia  OMIM 137192 |
| Tang, 2008 [57] | German | HCN2 | c.858T>C | TaqMan | 197/462 | No | 8 | PB+GC | 19p13.3 | No associated |
|  |  |  | c.1239G>C |  |  | No |  |  |  |  |
|  |  |  | c.1584+7C>T |  |  | No |  |  |  |  |
|  |  |  | c.1825+22_1825+42del |  |  | No |  |  |  |  |
|  |  |  | c.1990+24_1990+57dup |  |  | No |  |  |  |  |
|  |  |  | rs10408159 |  |  | No |  |  |  |  |
|  |  |  | rs4919872 |  |  | No |  |  |  |  |
| Rozycka, 2009 [58] | Polish | CHRNA4 | c.555C>T | PCR-RFLP | 92/137 | No | 12 | PB | 20q13 | other epilepsy  OMIM 118504 |
|  |  |  | 594C>T |  | 92/137 | No |  |  |  |  |
|  |  |  | 1674(+11)C>T |  | 92/222 | Yes |  |  |  |  |
|  |  |  | 1674(+14)A>G |  | 92/137 | No |  |  |  |  |
| Bai, 2009 [59] | Mexican, USA, Honduran | EFHC1 | rs3804506 475 C>T | PCR | 130/604 | No | 11 | PB+FB | 6p21 | JME  OMIM 608815 |
|  |  |  | rs3804505 545G>A |  | 130/604 | No |  |  |  |  |
|  |  |  | rs1266787 1343T>C |  | 130/614 | No |  |  |  |  |
|  |  |  | rs17851770 1855A>C |  | 130/552 | No |  |  |  |  |
| Layouni, 2010 [60] | European and Tunisian | BRD2 | rs3918149_A | Taqman | 159/154 | No | 11 | PB | 6p21 | JME  OMIM 608816 |
|  |  |  | rs516535_C |  |  | No |  |  |  |  |
|  |  |  | rs206781_T |  |  | No |  |  |  |  |
|  |  | TAP-1 | Ile333Val | PCR/SSCP |  | No |  |  | 6p21 | JME  OMIM 608816 |
|  |  |  | Asp637Gly |  |  | No |  |  |  |  |
| Layouni, 2010 [61] | Tunisian | TAP-1 | I333V | PCR/SSCP | 16/81 | No | 9 | PB | 6p21 | JME  OMIM 608816 |
|  | European |  | D637G |  | 154/159 | Yes |  |  |  |  |
| Muhle, 2010 [62] | German | GRM4 | rs2499669 G/A | SNPlex™ Genotyping System | - | No | 11 | PB | 6p21 | JME  OMIM 608816 |
|  |  |  | rs4711374 T/C |  | 214/732 | Yes |  |  |  |  |
|  |  |  | rs1466650 T/A |  | 214/731 | Yes |  |  |  |  |
|  |  |  | rs10947476 T/C |  | - | No |  |  |  |  |
|  |  |  | rs2499694 G/A |  | - | No |  |  |  |  |
|  |  |  | rs937039 G/A |  | - | No |  |  |  |  |
|  |  |  | rs2499697 C/A |  | - | No |  |  |  |  |
|  |  |  | rs1994582 T/C |  | - | No |  |  |  |  |
|  |  |  | rs2451357 G/A |  | - | No |  |  |  |  |
|  |  |  | rs9380405 T/C |  | 214/732 | Yes |  |  |  |  |
|  |  |  | rs745501 T/A |  | - | No |  |  |  |  |
|  |  |  | rs2499714 T/C |  | - | No |  |  |  |  |
|  |  |  | rs7763695 T/C |  | - | No |  |  |  |  |
|  |  |  | rs11753413 T/C |  | 215/729 | Yes |  |  |  |  |
|  |  |  | rs9469718 G/A |  | - | No |  |  |  |  |
|  |  |  | rs2451334 T/C |  | - | No |  |  |  |  |
|  |  |  | rs2029461 G/A |  | 215/732 | Yes |  |  |  |  |
| Gitaí, 2012 [63] | Northeast Brazil | GABRG2 | rs211037 | PCR/RFLP | 98/130 | No | 13 | PB | 5q34 | other epilepsy  OMIM 137164 |
| Balan, 2013 [64] | Indian | GABRA1 | rs2279020 | PCR | 201/267 | No | 12 | PB | 5q34 | JME  OMIM137164 |
|  |  | GABRA6 | rs3219151 |  |  | No |  |  |  |  |
|  |  | GABRB2 | rs2229944 |  |  | No |  |  |  |  |
|  |  | GABRG2 | rs211037 |  |  | Yes |  |  |  |  |
| Balan, 2013 [65] | South India | MVP | rs4788187 | PCR | 201/213 | No | 12 | PB | 16p12.1-p11.2 | No associated |
|  |  |  | rs3815824 |  |  | No |  |  |  |  |
|  |  |  | rs3815823 |  |  | No |  |  |  |  |
| Rozycka, 2013 [66] | Poland | CHRFAM7A | rs67158670 (delTG) | TaqMan | 167/258 | Yes | 8 | PB | 15q13-q14 | JME  OMIM 604827 |
|  |  |  | rs10649395 (insGTT) |  |  | No |  |  |  |  |
| Parihar, 2014 [67] | Indian | GRM4 | rs2029461 | PCR-RFLP | 249/186 | Yes | 12 | PB+FB | 6p21 | JME  OMIM 608816 |
|  |  |  | rs937039 |  |  | No |  |  |  |  |
|  |  |  | rs2499697 |  |  | No |  |  |  |  |
|  |  |  | rs745501 |  |  | No |  |  |  |  |
|  |  |  | rs2451334 |  |  | No |  |  |  |  |
| Neubauer, 2014 [68] | German | KCNQ2 | rs1801545 (c.1680C>G) | TaqMan | 234/457 | Yes | 10 | PB | 20q13.33 | Other epilepsy  OMIM 602232 |
|  |  | KCNQ3 | c.1945C>T (p.Pro574Ser) |  |  | Yes |  |  | 8q24.22 | Other epilepsy  OMIM 602235 |
| Esmail, 2014 [69] | Egyptian | 5HTT | VNTR | qRT-PCR | 40/40 | Yes | 8 | PB | 17q11.2 | No associated |
| Santos, 2014 [70] | Northeast Brazil | PER2 | rs2304672 | PCR-RFLP | 97/212 | No | 13 | PB | 2q37.3 | No associated |
|  |  | CLOCK | rs1801260 | PCR-RFLP |  | No |  |  | 4q18 | Other epilepsy  OMIM 611630 |
|  |  | PER3 | rs57875989 | PCR |  | No |  |  | 1p36.23 | No associated |
| Balan, 2014 [71] | South India | ABCB1 | rs1045642 (C3435T) | PCR-RFLP | 201/275 | Yes | 11 | PB | 7q21 | No associated |
|  |  |  | rs2032582 (G2677T) |  |  | No |  |  |  |  |
|  |  |  | rs1128503 (C1236T) |  |  | No |  |  |  |  |
|  |  |  | rs3213619 (T129C) |  |  | No |  |  |  |  |
|  |  |  | rs2214102 (21G/A) |  |  | No |  |  |  |  |
|  |  |  | rs1202168(+139C/T |  |  | No |  |  |  |  |
|  |  |  | rs1922242 (276T/A) |  |  | No |  |  |  |  |
|  |  | ABCG2 | rs2231142 (Gln141Lys; missense) |  |  | No |  |  | 4q22 | No associated |
|  |  |  | rs72552713 (Gln126Ter; stop gain) |  |  | No |  |  |  |  |
|  |  |  | rs2231137 (Val12Met; missense) |  |  | No |  |  |  |  |
| Born, 2015 [72] | Northeast Brazil | Prothrombin | rs1799963 | PCR-RFLP | 123/207 | No | 13 | PB | 11p11.2 | No associated |
| Guo, 2015 [73] | Chinese | KCNJ10 | rs1053074 | MassArray | 124/284 | No | 9 | PB | 1q23.2 | No associated |
|  |  |  | rs1130183 |  |  | No |  |  |  |  |
|  |  |  | rs12729701 |  |  | No |  |  |  |  |
|  |  |  | rs12402969 |  |  | No |  |  |  |  |
|  |  |  | rs1186688 |  |  | No |  |  |  |  |
|  |  |  | rs1186685 |  |  | No |  |  |  |  |
|  |  |  | rs6690889 |  |  | No |  |  |  |  |
|  |  |  | rs1890532 |  |  | No |  |  |  |  |
| Jiang, 2012 [74] | Chinese | SEZ-6 | c.1249dupC | PCR | 31/100 | No | 11 | PB | 17q11.2 | No associated |
|  |  |  | p.Thr546Ala |  |  | No |  |  |  |  |
|  |  |  | c.1807G > A |  |  | No |  |  |  |  |
| Le Hellard, 1999 [75] | European | HLA | HLA-DR13 (DRB1*1301) | SSP-PCR | 93/93 | No | 7 | PB | 6p21 | JME  OMIM 608816 |
|  |  |  | HLA-DR13 (DRB1*1302) |  |  | - |  |  |  |  |
|  |  |  | HLA-DR13 (DRB1*1301+*1302) |  |  | No |  |  |  |  |
|  |  |  | HLA-DQB1 (*0603) | PCR |  | - |  |  |  |  |
|  |  |  | HLA-DQB1 (*0604) |  |  | No |  |  |  |  |
|  |  |  | HLA-DQB1 (*0603+*0604) |  |  | No |  |  |  |  |
| Qu, 2015 [76] | Chinese | ATP1A3 | rs8107107 | MassArray | 124/284 | No | 10 | PB | 1q23.2 | No associated |
| Cavalleri, 2007 [77] | Irish | GABRR2 | rs9362632 | Illumina GoldenGate platform | 75/1118 | Yes | 11 | PB | 6q15 | No associated |
|  | UK |  |  |  | 41/1118 | No |  |  |  |  |
|  | Australian |  |  |  | 122/1118 | No |  |  |  |  |
|  | Combined (Irish, UK and Australian) |  |  |  | 238/1118 | Yes |  |  |  |  |
|  | Irish |  | rs7762767 |  | 75/1118 | Yes |  |  |  |  |
|  | UK |  |  |  | 41/1118 | No |  |  |  |  |
|  | Australian |  |  |  | 122/1118 | No |  |  |  |  |
|  | Combined (Irish, UK and Australian) |  |  |  | 238/1118 | Yes |  |  |  |  |
| Sapio, 2015 [78] | French | CPA6 | rs10957393 (c.133 T>C, p.Phe45Leu) | PCR | 124/238 | No | 10 | PB | 8q13.2 | other epilepsy  OMIM 609562 |
|  |  |  | rs17853192 (c.518 C>G, p.Ser173Cys) |  | 123/237 | No |  |  |  |  |
|  |  |  | rs17343819 (c.746 A>G, p.Asn249Ser) |  | 121/238 | No |  |  |  |  |
| Heron, 2007 [79] | Australian | GABRB3 | c.1-897T/C | PCR–RFLP | 44/180 | No | 5 | PB | 15q12 | other epilepsy  OMIM 137192 |
| Roshan, 2014 [80] | South India | LGI4 | G-to-N | PCR/SSCP | 75/100 | Yes | 9 | PB | 19q13.12 | No associated |
| Moen, 1995 [81] | Scandinavian | HLA | DQAI*0101 | PCR/SSO | 24/129 | No | 6 | PB | 6p21 | JME  OMIM 608816 |
|  |  |  | DQAI*0102 |  |  | No |  |  |  |  |
|  |  |  | DQAI*0103 |  |  | No |  |  |  |  |
|  |  |  | DQAI*0201 |  |  | No |  |  |  |  |
|  |  |  | DQAI*030X§ |  |  | No |  |  |  |  |
|  |  |  | DQAI*0401 |  |  | No |  |  |  |  |
|  |  |  | DQAI*050111 |  |  | No |  |  |  |  |
|  |  |  | DQAI*0601 |  |  | No |  |  |  |  |
|  |  |  | DQBl*0501 |  |  | No |  |  |  |  |
|  |  |  | DQBl*0502 |  |  | No |  |  |  |  |
|  |  |  | DQBI*05031 |  |  | No |  |  |  |  |
|  |  |  | DQBl*05032 |  |  | No |  |  |  |  |
|  |  |  | DQBI*0601 |  |  | No |  |  |  |  |
|  |  |  | DQBI*0602 |  |  | No |  |  |  |  |
|  |  |  | DQBI*0603 |  |  | No |  |  |  |  |
|  |  |  | DQBI*0604,5 |  |  | No |  |  |  |  |
|  |  |  | DQBI*0201 |  |  | No |  |  |  |  |
|  |  |  | DQBI*0301 |  |  | No |  |  |  |  |
|  |  |  | DQBI*0302 |  |  | No |  |  |  |  |
|  |  |  | DQBI*0303X |  |  | No |  |  |  |  |
|  |  |  | DQBI*0401 |  |  | No |  |  |  |  |
|  |  |  | DQBl*0402 |  |  | No |  |  |  |  |
|  |  |  | DRBI*I~ |  |  | Yes |  |  |  |  |
|  |  |  | DRBI*2 |  |  | No |  |  |  |  |
|  |  |  | DRBI*3 |  |  | No |  |  |  |  |
|  |  |  | DRBI*4 |  |  | No |  |  |  |  |
|  |  |  | DRBI*5 |  |  | No |  |  |  |  |
|  |  |  | DRBI*6 |  |  | No |  |  |  |  |
|  |  |  | DRBI*7 |  |  | No |  |  |  |  |
|  |  |  | DRBI*8 |  |  | No |  |  |  |  |
|  |  |  | DRBI*9 |  |  | No |  |  |  |  |
|  |  |  | DRBI*10 |  |  | No |  |  |  |  |
| Pal, 2003 [83] |  | HLA-DOB | rs11244 | Fluorescence polarization analysis | 20/64 | No | 7 | PB+FB | 6p21 | JME  OMIM 608816 |
|  |  | ABCB3 (TAP2) | rs241441 |  |  | No |  |  |  |  |
|  |  | ABCB2 (TAP1) | rs1057141 |  |  | No |  |  |  |  |
|  |  | PSMB9 | rs1044244 |  |  | No |  |  |  |  |
|  |  | LOC100294145 | rs241412 |  |  | No |  |  |  |  |
|  |  | HLA-DMB | rs10751 |  |  | No |  |  |  |  |
|  |  | HLA-DMB | rs2071556 |  |  | Yes |  |  |  |  |
|  |  | BRD2 | rs206787 |  |  | Yes |  |  |  |  |
|  |  | BRD2 | rs3918149 |  |  | Yes |  |  |  |  |
|  |  | BRD2 | rs620202 |  |  | No |  |  |  |  |
|  |  | BRD2 | rs516535 |  |  | Yes |  |  |  |  |
|  |  | BRD2 | rs635688 |  |  | Yes |  |  |  |  |
|  |  | BRD2 | rs2066741 |  |  | Yes |  |  |  |  |
|  |  | BRD2 | rs206781 |  |  | No |  |  |  |  |
|  |  | ---- | rs206777 |  |  | Yes |  |  |  |  |
|  |  | ---- | rs497058 |  |  | Yes |  |  |  |  |
|  |  | HLA-DNA or HLA-DOA | rs1044429 |  |  | No |  |  |  |  |
|  |  | HLA-DNA | rs2581 |  |  | No |  |  |  |  |
|  |  | HLA-DNA | rs365066 |  |  | No |  |  |  |  |
|  |  | HLA-DNA | rs375256 |  |  | No |  |  |  |  |
| **GWAS study** |  |  |  |  |  |  |  |  |  |  |
| Steffens, 2012 [82] | European | CHRM3 | rs12059546 | GWAS | 382/382 | Yes | 12 | PB+FB | 1q43 | No associated |
|  |  |  | rs1110615 |  |  | No |  |  |  |  |
|  |  | BSN | rs62261251 |  |  | No |  |  | 3q21.31 | No associated |
|  |  | MAST4 | rs39861 |  |  | No |  |  | 5q12.3 | No associated |
|  |  | --- | rs17669194 |  |  | No |  |  | 13q13.2 | No associated |

**Abbreviations:** PB: Population-based; FB: Family-based association study; GC: Genomic Control; ABCB1, ATP binding cassette subfamily B member 1; ABCG2, ATP binding cassette subfamily G member 2 (Junior blood group); ALDH5A1, aldehyde dehydrogenase 5 family member A1; ATP1A3, ATPase Na+/K+ transporting subunit alpha 3; BSN, bassoon presynaptic cytomatrix protein; BRD2, Bromodomain Containing 2; CHRNA4, cholinergic receptor, nicotinic alpha 4; CX36, connexin-36; GRM4, glutamate receptor, metabotropic 4; CACNA1A, calcium channel, voltage-dependent, P/Q type, alpha 1A subunit; CLOCK, clock circadian regulator; CHRFAM7A, CHRNA7 (cholinergic receptor, nicotinic, alpha 7, exons 5-10) and FAM7A (family with sequence similarity 7A, exons A-E) fusion; CHRM3, cholinergic receptor, muscarinic 3; CPA6, carboxypeptidase A6; DHPLC, Denaturing high pressure liquid chromatography; EFHC1, EF-hand domain (C-terminal) containing 1; EFHC2, EF-hand domain (C-terminal) containing 2; GABA, gamma-aminobutyric acid; GABABR1, gamma-aminobutyric acid (GABA) B receptor, 1; GABRG2, gamma-aminobutyric acid (GABA) A receptor, gamma 2; GABRA5, gamma-aminobutyric acid (GABA) A receptor, alpha 5; GABRB3, gamma-aminobutyric acid (GABA) A receptor, beta 3; GABRD, gamma-aminobutyric acid (GABA) A receptor, delta; GLRA3, glycine receptor alpha 3; GRM4, glutamate receptor, metabotropic 4; GWAS, genome-wide association study; HCN2, hyperpolarization activated cyclic nucleotide gated potassium channel 2; hKCa3, potassium channel, calcium activated intermediate/small conductance subfamily N alpha, member 3 (KCNN3); hSKCa3, potassium channel, calcium activated intermediate/small conductance subfamily N alpha, member 3 (KCNN3); HLA-DOA: major histocompatibility complex, class II, DO alpha (Also known as HLA-DNA); HLA-DOB: major histocompatibility complex, class II, DO beta; HLA-DMB: major histocompatibility complex, class II, DM beta; HLA-DQB1, major histocompatibility complex, class II, DQ beta 1; HLA-DRB1, major histocompatibility complex, class II, DR beta 1; JME, Juvenile Mioclonic Epilepsy; KCNJ3, potassium channel, inwardly rectifying subfamily J, member 3; KCNJ6, potassium channel, inwardly rectifying subfamily J, member 6; KCNJ10, potassium channel, inwardly rectifying subfamily J, member 10; KCNQ2, potassium channel, voltage gated KQT-like subfamily Q, member 2; KCNQ3, potassium channel, voltage gated KQT-like subfamily Q, member 3; LGI4: leucine-rich repeat LGI family member 4; LOC100294145: uncharacterized, Gene ID: 100294145, updated on 6-Oct-2016; MAOA, monoamine oxidase A; MVP, major vault protein; MAST, SPG21 - spastic paraplegia 21 (autosomal recessive, Mast syndrome); PAX6, paired box 6; PCR, Polymerase Chain Reaction; PCR-SSO, Polymerase Chain Reaction - Sequence-Specific Oligonucleotide; PCR-RFLP, Polymerase Chain Reaction-Restriction Enzyme Fragment Length Polymorphism; PCR-SSCP, Polymerase Chain Reaction - Single-Strand Conformation Polymorphism; PCR-SSCA, Polymerase Chain Reaction Single-Strand Conformation Analysis; PER2, period circadian clock 2; PER3, period circadian clock 3; PSMB9: proteasome subunit beta 9; SEZ6, seizure related 6 homolog (mouse); SSP-PCR, Single Specific Primer-Polymerase Chain Reaction; TAP1, transporter 1, ATP-binding cassette; USA, United States of America; TAP2: transporter 2, ATP binding cassette subfamily B member; VNTR, Variable Number Tandem Repeat; 5-HTT, serotonin transporter.
